# Deficiency of G9a Inhibits Cell Proliferation and Activates Autophagy via Transcriptionally Regulating c-Myc Expression in Glioblastoma

**DOI:** 10.3389/fcell.2020.593964

**Published:** 2020-11-27

**Authors:** Xiao Xue Ke, Rui Zhang, Xi Zhong, Lei Zhang, Hongjuan Cui

**Affiliations:** ^1^State Key Laboratory of Silkworm Genome Biology, College of Sericulture, Textile and Biomass Sciences, Southwest University, Chongqing, China; ^2^Cancer Center, Medical Research Institute, Southwest University, Chongqing, China; ^3^Chongqing Engineering and Technology Research Centre for Silk Biomaterials and Regenerative Medicine, Southwest University, Chongqing, China; ^4^Engineering Research Center for Cancer Biomedical and Translational Medicine, Southwest University, Chongqing, China

**Keywords:** glioblastoma, cell proliferation, autophagy, G9a, c-Myc

## Abstract

Glioblastoma is an aggressive and difficult to treat cancer. Recent data have emerged implicating that histone modification level may play a crucial role in glioma genesis. The histone lysine methyltransferase G9a is mainly responsible for the mono- and di-methylation of histone H3 lysine 9 (H3K9), whose overexpression is associated with a more aggressive phenotype in cancer. However, the detailed correlations between G9a and glioblastoma genesis remain to be further elucidated. Here, we show that G9a is essential for glioblastoma carcinogenesis and reveal a probable mechanism of it in cell proliferation control. We found that G9a was highly expressed in glioblastoma cells, and knockdown or inhibition of G9a significantly repressed cell proliferation and tumorigenesis ability both *in vitro* and *in vivo*. Besides, knockdown or inhibition of G9a led to a cell cycle arrest in G2 phase, as well as decreased the expression of CDK1, CDK2, Cyclin A2, and Cyclin B1, while it induced the activation of autophagy. Further investigation showed that G9a deficiency induced cell proliferation suppression, and activation of autophagy was rescued by overexpression of the full-length c-Myc. Chromatin immunoprecipitation (ChIP) assay showed that G9a was enriched on the −2267 to −1949 region of the c-Myc promoter in LN-229 cells and the −1949 to −1630 region of the c-Myc promoter in U-87 MG cells. Dual-luciferase reporter assay showed that c-Myc promoter activity was significantly reduced after knockdown or inhibition of G9a. Our study shows that G9a controls glioblastoma cell proliferation by transcriptionally modulating oncogene c-Myc and provides insight into the capabilities of G9a working as a potential therapeutic target in glioblastoma.

## Introduction

Glioblastoma is one of the most aggressive and deadliest cancers with a uniformly poor patient prognosis (Silantyev et al., 2019). Traditional anti-cancer treatment is inefficient against glioblastoma due to its high proliferation ability and its extremely strong invasion capacity. Currently, standard care includes maximum surgical resection, followed by radiotherapy and simultaneous chemotherapy with temozolomide (TMZ) (Ozdemir-Kaynak et al., 2018). Although great advances have been obtained in diagnostic technology (Drake et al., 2020), surgery technique, and radio-/chemotherapy (Oberheim Bush et al., 2019), long-term survivors are very rare and tumors usually recur within a short period of time (Silantyev et al., 2019), and the 5-year survival is still lower than 6% (Shergalis et al., 2018). Therefore, novel therapeutic strategies of glioblastoma are urgently needed, and gaining a comprehensive understanding of the molecular mechanisms of glioblastoma initiation and progression is very important.

G9a was first discovered as a gene located in the major histocompatibility complex (MHC) locus in mice and human leukocyte antigen (HLA) locus in humans (Spies et al., 1989; Kendall et al., 1990; Milner and Campbell, 1993; Brown et al., 2001). Besides, considering its predominant localization, G9a was also defined as histone lysine methyltransferase (Tachibana et al., 2001) that is mainly responsible for the mono- and di-methylation of histone H3 lysine 9 (H3K9) (Tachibana et al., 2002, 2005). Recent data have emerged, implicating that histone modification level may play a crucial role in glioma genesis. H3K9me2, H3K9me3, H3K4me3, H3K27me2, H3K27me3, and H3K79me2 are reported to be associated with IDH1 mutation in gliomas (Lohmann et al., 2010; Xu et al., 2011; Lu et al., 2012; Turcan et al., 2012). However, besides its ability to methylate substrates, G9a has also been considered as a gene to have methyltransferase-independent activities that influence the expression of target gene (Lee et al., 2006; Purcell et al., 2011; Bittencourt et al., 2012). Thus, G9a may act as an important regulator in glioblastoma with its methyltransferase activity or methyltransferase-independent function.

It has been observed that the expression of G9a is upregulated in a number of cancers, including aggressive lung cancer, multiple myeloma, aggressive ovarian carcinoma, brain cancer, hepatocellular carcinoma, esophageal squamous cell carcinoma, and malignant melanoma (Wozniak et al., 2007; Gao et al., 2013; Hua et al., 2014; Lehnertz et al., 2014; Hu et al., 2019; Dang et al., 2020). Higher G9a expression levels have often been associated with poor prognosis (Chen et al., 2010; Ke et al., 2014; Zhang et al., 2019). G9a is significantly increased in colorectal cancer cells, which is associated with tumor progression and maintenance of malignancy. While silence of G9a in colorectal cancer cells induces increased DNA damage (Zhang et al., 2015). In mouse models of acute myeloid leukemia, loss of G9a blocks cell proliferation and retards disease process (Lehnertz et al., 2014). In hypoxic solid tumors, G9a was demonstrated to inhibit the expression of cell adhesion factors such as E-cadherin and epithelial cell adhesion molecules (Ep-cam), indicating that G9a might be a key factor in the occurrence of metastasis (Wozniak et al., 2007; Chen et al., 2010). The correlation between G9a and maintenance of the malignant phenotype suggests that targeting this enzyme might represent a novel strategy for the treatment of solid tumors, which is characterized by hypoxic regions and higher risk of metastasis (Casciello et al., 2015; Chen et al., 2017). Additionally, BIX01294, the inhibitor of G9a, is reported to sensitize glioma cells to TMZ (Ciechomska et al., 2018) and to induce autophagy-dependent differentiation of glioma stem-like cells (Ciechomska et al., 2016). In this study, we investigated the role of G9a in maintenance of the malignant phenotype in glioblastoma. Our results reveal that G9a modulate glioblastoma cell proliferation by transcriptionally activate oncogene c-Myc.

## Materials and Methods

### Cell Culture

Human glioblastoma cell lines A172, U-87 MG, LN-229, and human normal astrocyte cell line SVGP12 were obtained from the American Type Culture Collection (ATCC; Manassas, VA, USA) and maintained in Dulbecco's modified Eagle's medium (DMEM) supplemented with 10% fetal bovine serum (FBS) plus 1% penicillin and streptomycin (P/S). The lentiviral packaging cell line 293FT was cultured in DMEM containing 10% FBS, 0.1 mM MEM non-essential amino acids, 1 mM MEM sodium pyruvate, 4 mM L-glutamine, 1% P/S, and 0.5 mg/ml G418. All cells were cultured at 37°C in a humidified incubator with 5% CO_2_. All the growth media, FBS, and supplemental reagents were obtained from Invitrogen®, Life Technologies (Thermo Fisher Scientific, Inc., Waltham, MA, USA).

### Lentiviral Constructs and Infection

The lentiviral constructs pLK0.1-puro-shG9a and pLK0.1-puro-shGFP were obtained from Dr. Han-Fei Ding (Georgia Regents University, Augusta, GA, USA) as a gift. The lentiviral constructs were transfected into 293FT packaging cells using Invitrogen® Lipofectamine® 2000 reagent (Thermo Fisher Scientific, Inc.). Then, virus-containing supernatants were harvested and tittered and used to infect the target cells with 4 μg/ml Polybrene (Santa Cruz Biotechnology, Inc., Dallas, TX, USA). One day after the final round of infection, the target cells were cultured in the presence of 2 μg/ml puromycin (Life Technologies; Thermo Fisher Scientific, Inc.) for 3 days. Finally, puromycin-resistant cells were pooled.

### Inhibitor Treatment

LN-229 and U-87 MG cells were seeded and cultured in six-well plates at a concentration of 1 × 10^5^ cells/well. The G9a inhibitor BIX01294 (BIX) was used as descried before (Ke et al., 2014). Chloroquine (CQ) was used at the concentration of 3.5 μM combined with BIX, and the treatment of CQ was executed by 6 h (Klionsky et al., 2016). BIX and CQ are both purchased from Sigma-Aldrich, Merck KGaA, Darmstadt, Germany.

### Cell Proliferation and Flow Cytometry Assays

For cell proliferation assays, cells were collected at different time points and analyzed by CCK-8 (CK04-05, DOJINDO, Kamimashiki gun, Kumamoto, Japan). For cell counting, all adherent and floating cells were pooled, then stained by trypan blue dye (#145-0021, Bio-Rad, Hercules, CA, United States), and analyzed with the TC10 Automated Cell Counter (Bio-Rad). For cell cycle assay, cell samples were harvested and washed twice with ice-cold phosphate-buffered saline (PBS), then fixed with 70% ethanol, stained with propidium iodide (PI), and analyzed by flow cytometry (BD FACS C6, BD BioSciences, San Jose, CA, United States). For cell apoptosis assay, all adherent and floating cells were harvested by centrifugation and then samples were determined by the Annexin V-FITC kit (Sigma), using flow cytometry (BD FACS C6, BD BioSciences). The data were analyzed with CellQuest Pro software (BD BioSciences).

### Real-Time qPCR Assay

Cell samples were harvested and lysed by Trizol (Invitrogen®, Life Technologies) to extract total RNA, which was reverse transcribed into cDNA by M-MLV (Promega, Promega Corporation, Madison, WI, United States). Then, MMP1 and N-cadherin mRNA transcript was determined by real-time qPCR, using the Eastep® qPCR Master Mix (Promega). The real-time qPCR assay was performed in triplicate and carried out by using the LightCycler® 96 real-time PCR system (Roche, F. Hoffmann-La Roche Ltd., Basel, Switzerland). All individual values were normalized to that of the GAPDH control.

### Western Blot Analysis

Human glioblastoma cells or tumor tissues were harvested and washed with ice-cold PBS. Then, samples were suspended in SDS sample buffer, boiled for 10 min, and centrifuged at 12,000 rpm for 15 min. Thirty micrograms of protein samples were separated using 8–12% SDS-polyacrylamide gel electrophoresis (SDS-PAGE) and transferred to a PVDF membrane (Millipore Corporation). The PVDF membrane was blocked with 5% milk for 1 h and then incubated with a primary antibody at 4°C overnight. Subsequently, the PVDF membrane was probed with the secondary antibody and visualized by enhanced chemiluminescence (ECL) (Beyotime Institute of Biotechnology, Haimen, China). The primary antibodies were as follows: anti-G9a (1:500, Santa Cruz), anti-a-tubulin (1:2000, Sigma), anti-CDK1 (1:1000, Abcam Cambridge, MA, United States), anti-CDK2 (1:500, Santa Cruz), anti-Cyclin A2 (1:2000, Abcam), anti-Cyclin B1 (1:3000, Abcam), anti-MMP1 (1:1000, Abcam), anti-N-cadherin (1:5000, Abcam), anti-LC3B (1:1000, Cell Signaling Technologies, Danvers, MA, United States), anti-p62 (1:1000, Abcam), anti-c-Myc (1:1000, Abcam), and anti-H3K9me2 (1:2000, Abcam). Horseradish peroxidase-conjugated goat anti-mouse IgG (1:20,000), goat anti-rabbit IgG (1:20,000), and rabbit anti-goat IgG (1:10000) (KPL, Gaithersburg, Maryland, United States) were used as secondary antibodies.

### Immunofluorescence Staining

LN-229 and U-87 MG cells were seeded and cultured in 24-well plates at a concentration of 2 × 10^3^ cells/well. For immunofluorescent staining, cells were washed with PBS and fixed in 4% paraformaldehyde (PFA) in PBS for 20 min at room temperature. Subsequently, cells were permeabilized with 0.3% Triton X-100 for 5 min and blocked with 10% goat serum for 1 h, then incubated with a primary antibody at 4°C overnight. The next day, cells were incubated with the appropriate secondary antibody for 2 h; 300 nM 4′,6-diamidino-2-phenylindole (DAPI) in PBS was used for nuclear staining. The primary antibodies were used at a dilution of 1:500 for the rabbit monoclonal antibody against Ki67 (Abcam) and 1:200 for the rabbit monoclonal antibody against LC3B (Cell Signaling Technologies). Alexa Fluor 594 Goat Anti-Rabbit IgG (H+L) and Alexa Fluor 488 Goat Anti-Rabbit IgG (H+L) (Invitrogen, Carlsbad, CA, United States) were used as secondary antibodies. Cell pictures were photographed using an Olympus IX71 inverted fluorescence microscope or an Olympus FV1000 confocal microscope (Olympus, Tokyo, Japan).

### Soft Agar Clonogenic Assay

LN-229 and U-87 MG cells were mixed with 0.3% Noble agar in growth medium and seeded at 1,500 cells/well into six-well plates containing a solidified bottom layer (0.6% Noble agar in the same growth medium). After 21 to 28 days of culture, colonies were stained with 5 mg/ml 3-(4,5-dimethylthiazol-2-yl)-2,5-diphenyltetrazolium bromide (MTT) for 15 min and then photographed and recorded by microscope (Olympus CKX41, Olympus).

### Migration, Invasion, and Wound Healing Assay

For the migration and invasion assay, cells were seeded in the upper chambers (8 μm pore size; Corning, Beijing, China) of a 24-well Boyden transwell chamber. The membranes were coated with Matrigel (BD Biosciences) in the invasion assay. Cells seeded in the upper chamber were cultured in serum-free media, while media supplemented with 10% FBS was used as a chemoattractant and added into the lower chamber. After 48 h of culture, cells were fixed in 4% PFA and stained with crystal violet. Then, cells were counted under a bright-field microscopy (Olympus CKX41, Olympus). The mean number of cells were calculated from 10 randomly chosen microscopic fields per filter.

For the wound healing assay, cells were seeded in six-well plates and cultured to grow to full confluence. Then, wounds were made in the middle of the well by using a 10-μl pipette tip. The wound healing process was monitored under a microscope (Olympus CKX41, Olympus).

### *In vivo* Tumorigenic Assay

Four-week-old female non-obese diabetic severe combined immunodeficient (NOD/SCID) mice were used in xenograft assay. The mice were housed in the animal facility of Southwest University (Chongqing, China) under specific pathogen-free (SPF) conditions and maintained under constant temperature and humidity.

In orthotopic transplantation experiments, six mice were used in each group. Human glioblastoma cell lines (LN-229 and U-87 MG) (1 × 10^5^ cells) stably transfected with shGFP, shG9a, shG9a/GFP, and shG9a/c-Myc, respectively, were slowly injected into the brain of each mouse. Intracerebral injection was performed with the following coordinates: 2 mm to the right of the bregma, 1 mm anterior to the coronal suture, 3 mm from the underside of the skull. Five microliters of cell suspension was injected into the injection site using a 10-μl Hamilton syringe (Hamilton Co., Reno, NV, United States) over a 5-min period. Upon completing injection, the syringe needle was left in place for another 3 min and then slowly removed (1 min withdraw 1 mm). All mice postoperatively were monitored until recovery from the anesthesia. At the termination of the animal experiment, all brains were collected and analyzed by Western blotting, hematoxylin and eosin (H&E) staining, and immunohistochemical analysis. Randomization and single blinding were used for measurement. Based on animal welfare, all surviving mice were euthanized on the 30th day after injection.

In the experiment of subcutaneous xenograft, a total of 10 mice were used. A total of 1 × 10^6^ U-87 MG cells were resuspended in 100 μl of DMEM and injected subcutaneously into the flanks of each mouse. After 1 week of tumor growth, the mice were randomly divided into two groups. One group was injected with BIX, and the other group was injected with water as control. At the termination of the experiment, the tumors were removed and weighed. All other details of the subcutaneous xenograft assay were described before (Ke et al., 2014).

### Histology and Immunohistochemistry

The brain tissues were embedded in paraffin blocks and cut into 4-μm slices and then stained with H&E. For immunohistochemical staining, the sections were deparaffinized, rehydrated, and then immersed in 10 mmol/L citrate buffer (pH 6.0) at 95°C for 20 min for antigen retrieval and subsequently washed in PBS. The endogenous peroxidase activity was quenched with 0.6% H_2_O_2_ in methanol, and the sections were blocked with normal goat serum. Then, the sections were sequentially incubated with primary antibodies (G9a, Ki67) and horseradish peroxidase-linked secondary antibodies. The sections were covered with 3,3′-diaminobenzidine (Sigma-Aldrich; Merck KGaA) for visualizing the immunostaining and then counterstained with hematoxylin before being examined using a light microscope (Nikon 80i, Nikon, Tokyo, Japan).

### Chromatin Immunoprecipitation Assay

The chromatin immunoprecipitation (ChIP) assay was performed by using a ChIP assay kit (Millipore) according to the manufacturer's instructions. Generally, at least 1 × 10^7^ LN-229 and U-87 MG cells were cross-linked by 1% formaldehyde and then lysed in ChIP lysis buffer. Chromatin DNA was sheared into 200- to 800-bp fragments by ultrasonication. Precleared chromatin was immunoprecipitated with G9a primary antibody (1:20, Abcam) or H3K9me2 primary antibody (1:25, Abcam), and then DNA was purified after reverse cross-linking for quantitative real-time PCR (qRT-PCR).

### Luciferase Reporter Assay

The c-Myc promoter fragment was amplified by PCR and ligated into the pGL3-basic vector, which was purchased from Promega (Promega Corporation, Madison, WI, United States). The sequence of the construct was confirmed by gene sequencing (BGI, Shenzhen, China). The empty pGL3-basic vector was used as a negative control. Cells were seeded in 24-well plates at a concentration of 2 × 10^5^ cells/well for cell transfection. A total of 1 μg of pGL3 plasmid and 100 ng of pRL-TK internal control vector (Promega) were co-transfected into 293FT cells in serum-free Opti-MEM Reduced Serum Medium (Life Technologies; Thermo Fisher Scientific, Inc.). After 6 h of transfection, culture medium was added to each well up to a final volume of 1 ml. Subsequently, cells were further incubated for 48 h. Then, a luciferase reporter assay was performed according to the manufacturer's instructions (Promega). Luciferase activity was normalized to pRL-TK activity.

### Statistical Analysis

All experiments were carried out in triplicates independently, and quantitative data are expressed as the mean ± SD. A two-tailed Student's *t*-test was performed for paired samples. One- or two-way analysis of variance followed by Dunnett's or Bonferroni's multiple comparison were performed for comparing multiple groups. *P* < 0.05 was considered to indicate a statistically significant result. All calculations were performed using the SPSS software package 14.0 (SPSS, Inc., Chicago, IL, United States). GraphPad Prism 6 (GraphPad Software, Inc., La Jolla, CA, United States) was applied for chart drawing.

### Patient Data Analysis

Patient database analysis was carried out online from the UALCAN cancer database (Chandrashekar et al., 2017) (http://ualcan.path.uab.edu/index.html) and the R2: Genomics Analysis and Visualization Platform (http://r2.amc.nl). All prognosis analyses were conducted online. For UALCAN database, the chart was downloaded. For R2 database, all data and *P*-values (log-rank test) were downloaded and then the chart was performed using GraphPad Prism 6.

## Results

### G9a Is Required for Glioblastoma Cell Proliferation

**Figure 1:**
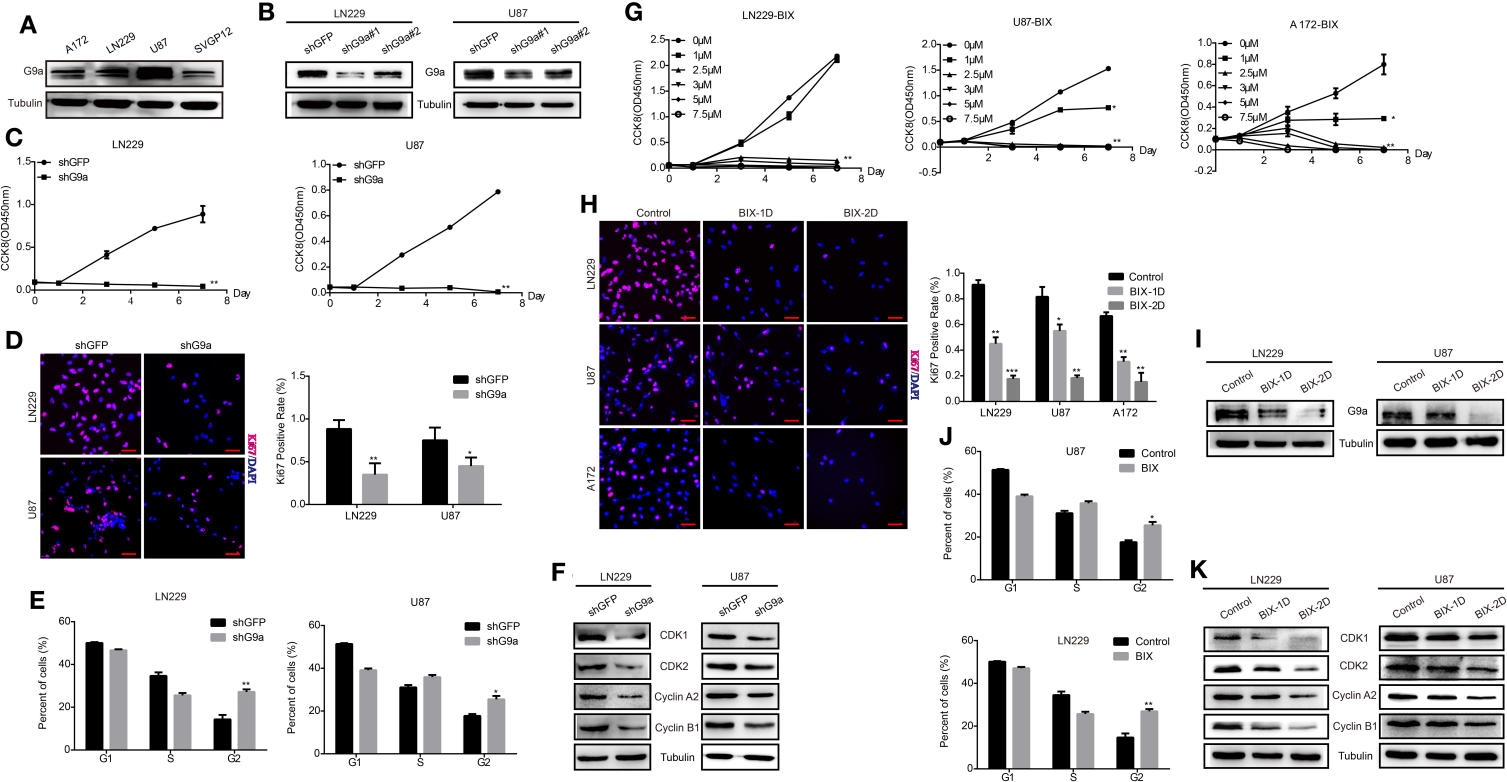
G9a is required for glioblastoma cell proliferation. **(A)** Western blot assay to check G9a expression in glioblastoma cells A172, LN-229, U-87 MG, and human normal astrocyte cell line SVGP12. **(B)** Western blot assay to check G9a expression level in LN-229 and U-87 MG cells after G9a knockdown by shRNA. **(C)** CCK-8 assay was performed to examine the effect of G9a knockdown on cell viability in LN-229 and U-87 MG cells. **(D)** Representative fluorescent micrographs of Ki67 assays and quantification of Ki67-positive cells of LN-229 and U-87 MG cells after G9a knockdown by shRNA. Scale bar, 20 μm. **(E)** Quantification of cell population in each cell cycle phase was analyzed by flow cytometry assay in LN-229 and U-87 MG cells after G9a knockdown by shRNA. **(F)** Western blot assay to check G2 cell cycle regulatory proteins in LN-229 and U-87 MG cells after G9a knockdown by shRNA. **(G)** CCK-8 assay was performed to examine the effect of G9a inhibitor treatment on cell viability in LN-229, U-87 MG, and A172 cells. **(H)** Representative fluorescent micrographs of Ki67 assays and quantification of Ki67-positive cells of LN-229, U-87 MG, and A172 cells after G9a inhibitor treatment. Scale bar, 20 μm. **(I)** Western blot assay to check G9a expression level in LN-229 and U-87 MG cells after G9a inhibitor treatment. **(J)** Quantification of cell population in each cell cycle phase was analyzed by flow cytometry assay in LN-229 and U-87MG cells after G9a inhibitor treatment. **(K)** Western blot assay to check G2 cell cycle regulatory proteins in LN-229 and U-87 MG cells after G9a inhibitor treatment. BIX-1D for BIX-treatment 1 day, BIX-2D for BIX-treatment 2 days. All experiments were carried out in triplicates independently, and all data are shown as the means ± s.d. *P < 0.05, **P < 0.01, ***P < 0.001. All *P*-values are based on control vs. treatment.

To investigate the role of G9a in glioblastoma cell proliferation, both specific inhibitor of G9a and lentivirus-induced gene knockdown were used in our experiments. Results of Western blot analysis confirmed that G9a was expressed in both glioblastoma cells and astrocyte cells (Figure 1A). The expression of G9a in glioblastoma cells was decreased after inhibitor treatment (Figure 1I) and G9a-shRNA-induced gene knockdown, and the no.1 shG9a had the higher effectivity in knockdown G9a expression (Figure 1B). Hence, for the following experiments, the no.1 shG9a was used as a representative G9a-knockdown means. As shown in Figure 1C, when G9a was downregulated in glioblastoma cells, cell proliferation was significantly inhibited. Cell proliferation rate was dropped obviously, and cell numbers of Ki67 (a well-known cell proliferation marker)-positive cells were evidently reduced (Figures 1C,D). Then, the cell cycle distribution of glioblastoma cells was analyzed by flow cytometry. Results showed that G9a knockdown induced cell cycle arrest at the G2 phase (Figure 1E and Supplementary Figure 1A). Similar results were observed in inhibitor-treatment cells (Figures 1G,H,J). According to the result shown in Figure 1G, 2.5 μM BIX was used in the following inhibitor treatment. To explain the molecular mechanism underlying G9a-knockdown-induced cell cycle arrest, we detected the expression of some G2 cell cycle regulatory proteins. We found that the expression of CDK1, CDK2, Cyclin A2, and Cyclin B1 was declined in both inhibitor-treatment cells and G9a-knockdown cells (Figures 1F,K). Collectively, these findings demonstrated that G9a was essential for glioblastoma cell proliferation.

### G9a Is Responsible for Glioblastoma Cell Migration and Invasion

**Figure 2:**
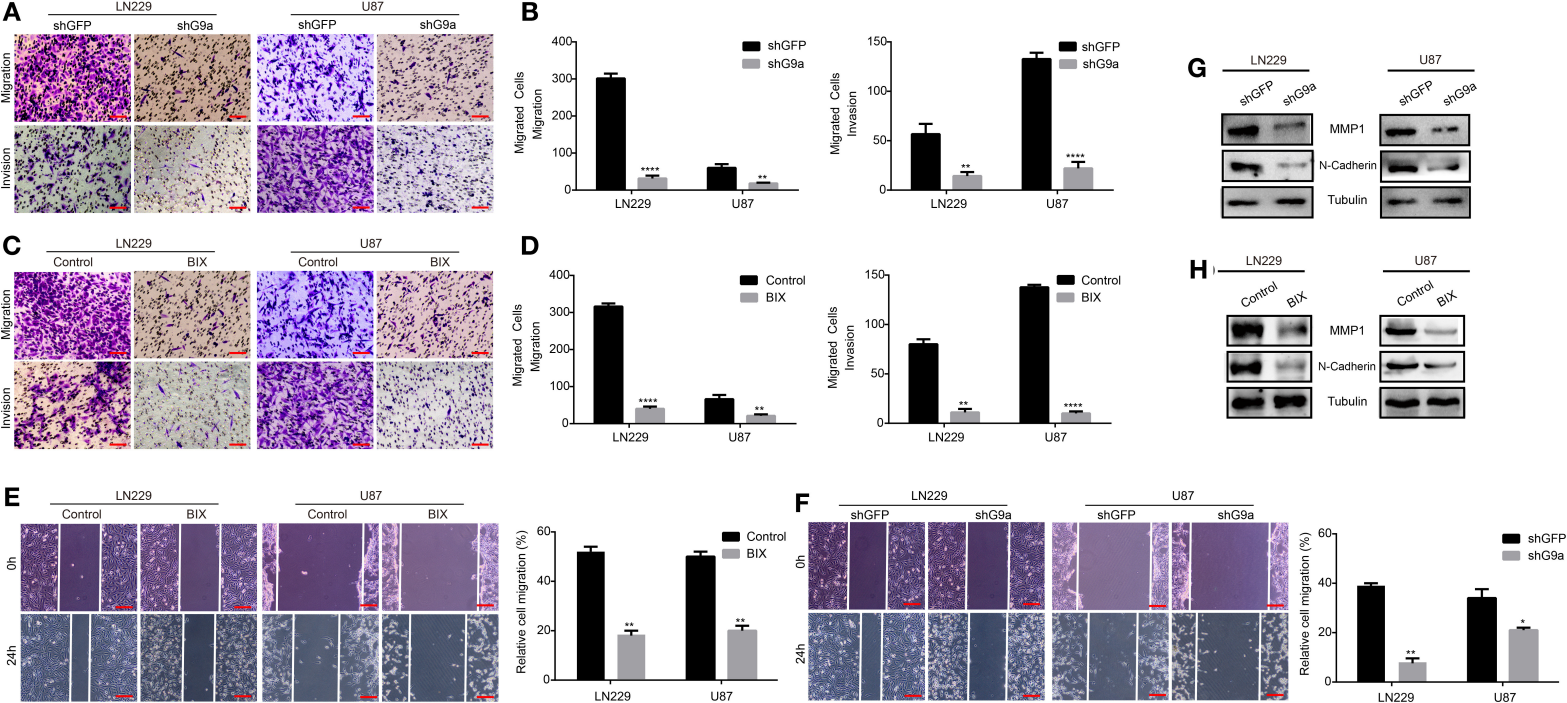
G9a is responsible for glioblastoma cell migration and invasion. **(A–D)** Migration assay and invasion assay were performed in LN-229 and U-87 MG cells after G9a knockdown or G9a inhibitor treatment. **(E,F)** Wound healing assay was performed in inhibitor-treated cells or G9a-knockdown cells. **(G,H)** Western blot analysis to characterize the expression of some key metastasis-related proteins in G9a-knockdown cells or inhibitor-treated cells. All experiments were carried out in triplicates independently, and all data are shown as the means ± s.d. *P < 0.05, **P < 0.01, ****P < 0.0001. All *P*-values are based on control vs. treatment.

As one of the most aggressive tumors, glioblastoma cells reveal powerful migration and invasion abilities. High expression of G9a was also observed in glioblastoma cells. To explore whether G9a was involved in glioblastoma migration and invasion, we performed migration assays and invasion assays. Results are shown in Figure 2. When LN-229 and U-87 MG cells were treated by specific inhibitor of G9a or G9a expression in LN-229 and U-87 MG cells was knocked down by shG9a, these glioblastoma cells migrated much slower than control cells (Figures 2A–D). In addition, wound healing assays were performed, and results demonstrated that the migratory capability was much weakened in inhibitor-treatment cells and G9a-knockdown cells (Figures 2E,F). Moreover, we performed molecular analyses to confirm the effect of G9a knockdown on cell migration and invasion. The expression of two key metastasis-related genes was examined by real-time PCR and Western blot assay. The result showed that inhibitor treatment and downregulation of G9a in LN-229 and U-87 MG cells significantly reduced the expression level of matrix metalloproteinase (MMP1) and a mesenchymal marker N-cadherin (Figures 2G,H, Supplementary Figure 1C). All these data indicated that G9a has a decisive impact on glioblastoma cell migration and invasion.

### G9a Is Essential for Glioblastoma Cell Clonogenicity *in vitro* and Tumorigenesis *in vivo*

**Figure 3:**
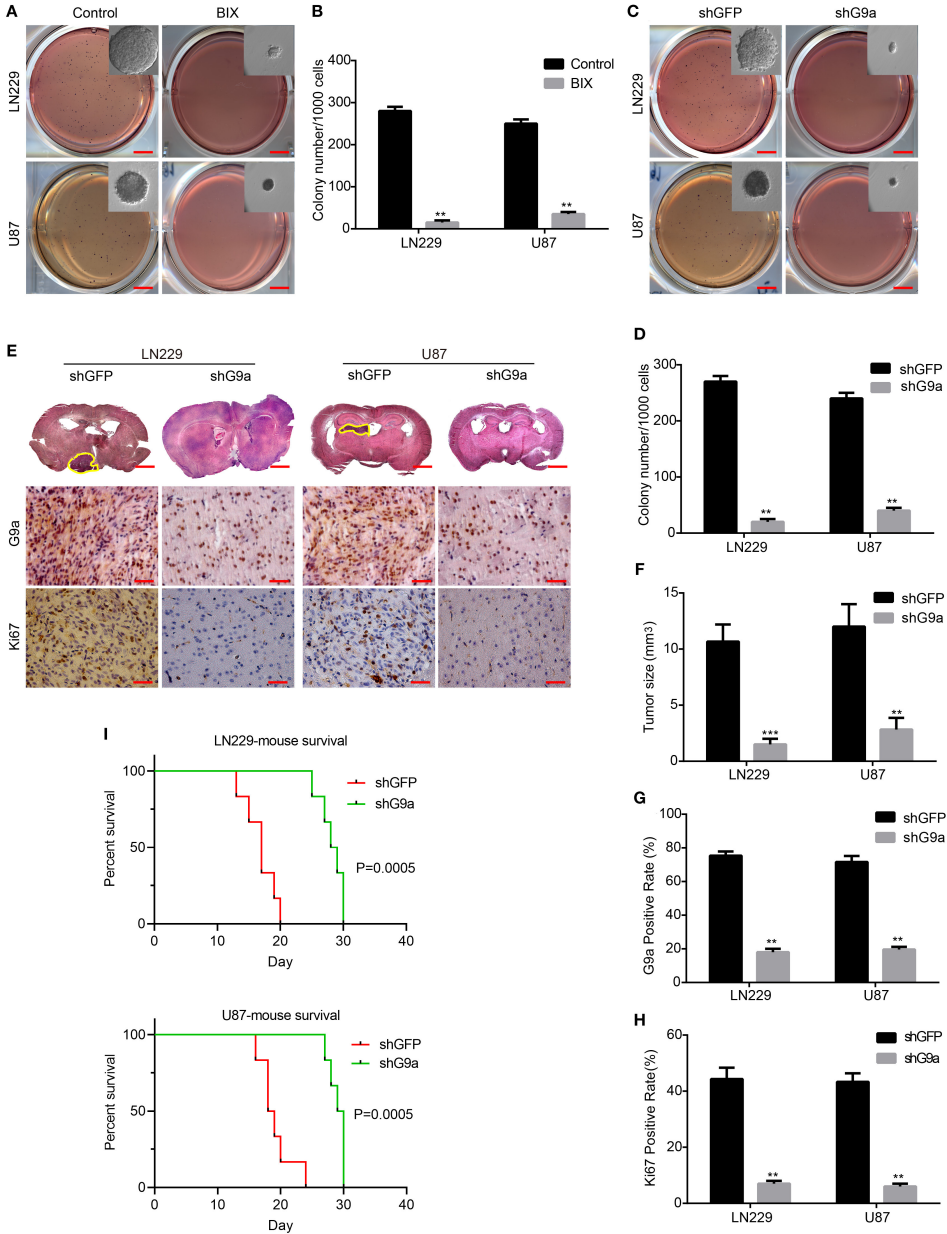
G9a is essential for glioblastoma cell clonogenicity *in vitro* and tumorigenesis *in vivo*. **(A–D)** The colony formation assay was performed in LN-229 and U-87 MG cells after G9a knockdown or G9a inhibitor treatment. **(E)** Orthotopic implantation was performed after G9a knockdown in LN-229 and U-87 MG cells. Representative images of the hematoxylin and eosin (H&E) staining (upper) and immunohistochemistry analysis of G9a expression (middle) and Ki67 expression (lower) are presented. **(F)** Quantification of effects of G9a knockdown on tumor size, **(G)** G9a expression, and **(H)** Ki67-positive cells in LN-229 and U-87 MG cells. **(I)** Survival curve of mice in orthotopic transplantation. All experiments were carried out in triplicates independently, and all data are shown as the means ± s.d. **P < 0.01, ***P < 0.001. All *P*-values are based on control vs. treatment.

To evaluate the effect of G9a on clonogenicity and tumorigenesis of glioblastoma cells, soft agar colony formation analyses, subcutaneous xenograft, and orthotopic implantation experiments on NOD/SCID mice were carried out. In the soft agar assays, the colonies in inhibitor-treatment groups and G9a-knockdown groups were much smaller and fewer than the corresponding control groups (Figures 3A–D). In the orthotopic implantation experiments, the tumor growth was evidently arrested in NOD/SCID mice injected with G9a-knockdown cells compared with the control mice (Figures 3E,F). The mouse survival was significantly extended by G9a knockdown (Figure 3I). Further immunohistochemical staining of the xenograft tumors exhibited that G9a expression was significantly decreased in the shG9a tumor sample; moreover, the expression of Ki67 was dropped in the G9a-knockdown tumor samples (Figures 3G,H). In addition, we also employed the subcutaneous transplantation to evaluate the effect of G9a inhibition on tumor growth, after tumors have already formed. Supplementary Figure 1F showed that the control group developed larger tumor masses, and BIX treatment significantly diminished the tumorigenic activity of U-87 MG cells during the same time period. These results suggested that G9a plays a critical role in clonogenicity and tumorigenesis of glioblastoma cells.

### G9a Deficiency Activates Autophagy of Glioblastoma Cells

**Figure 4:**
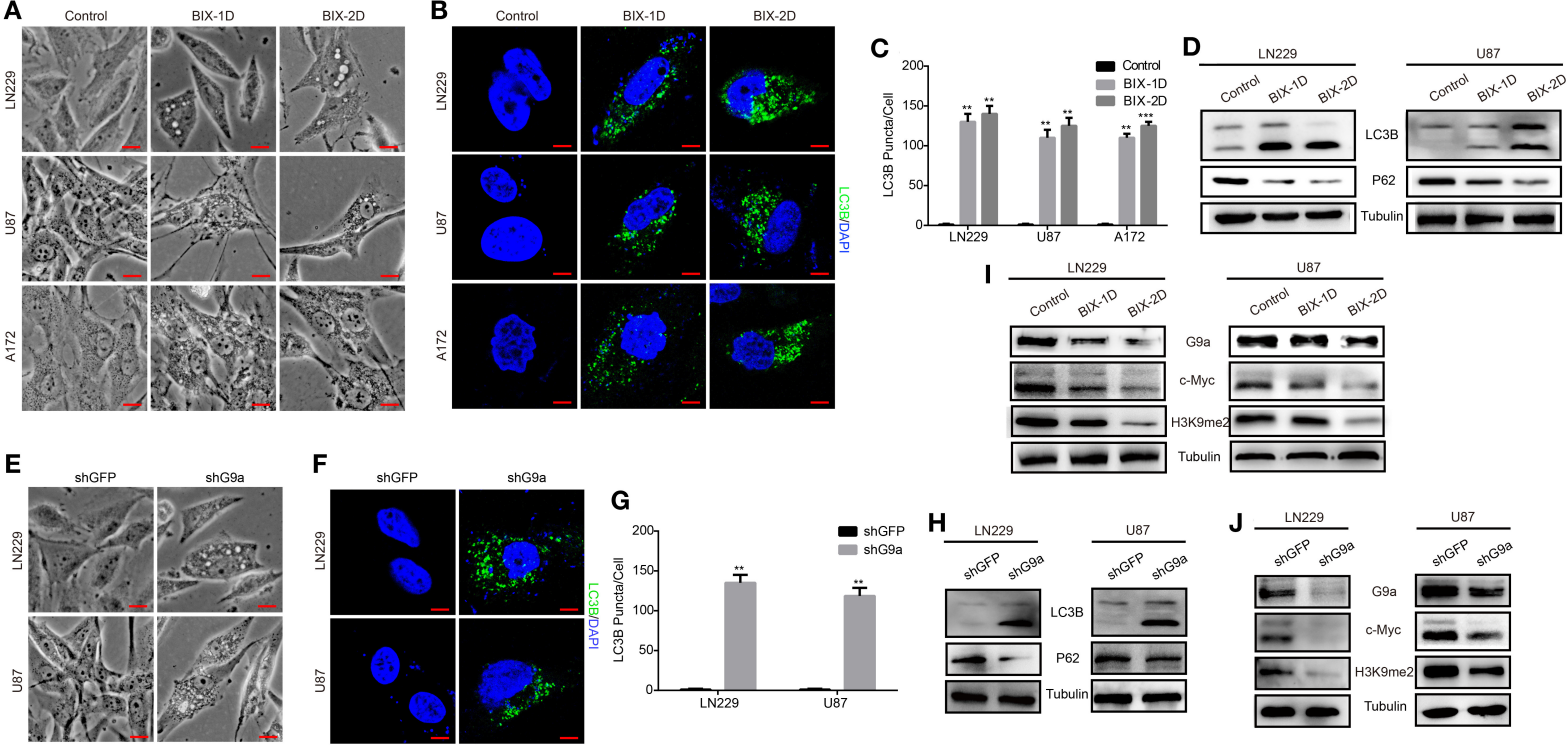
G9a deficiency activates autophagy of glioblastoma cells. **(A,E)** Micrograph of LN-229 and U-87 MG cells after G9a inhibitor treatment or G9a knockdown by shRNA. Scale bar, 10 μm. **(B,F)** Fluorescence micrograph of immunofluorescence assay of LC3B expression in inhibitor-treated cells or G9a-knockdown cells. Scale bar, 5 μm. **(C,G)** Quantification of LC3B puncta in inhibitor-treated cells or G9a-knockdown cells. **(D,H)** Western blot analysis of autophagy-related genes in inhibitor-treated cells or G9a-knockdown cells. **(I,J)** Western blot analysis to check the correlation of expression of G9a, c-Myc, and H3K9me2. BIX-1D for BIX-treatment 1 day, BIX-2D for BIX-treatment 2 days. All experiments were carried out in triplicates independently, and all data are shown as the means ± s.d. **P < 0.01, ***P < 0.001. All *P*-values are based on control vs. treatment.

According to our research, G9a has a crucial impact on glioblastoma cell proliferation, migration, and tumorigenesis. To further explore how G9a accelerated glioblastoma growth, we examined the molecular mechanism of the decrease in cell numbers observed after inhibitor treatment and downregulation of G9a. We found that when glioblastoma cells were treated by specific inhibitor of G9a, there are a large number of vacuoles arising in cells (Figure 4A). The expression of LC3B protein, an autophagy initiation marker, was increased markedly, while the expression of p62 protein was reduced (Figure 4D). In addition, increased conversion of LC3B-I to LC3B-II was demonstrated by Western blot analyses. Moreover, immunofluorescence staining was conducted and revealed that inhibitor-treatment cells showed a punctate pattern of LC3B fluorescence, representing the recruitment of LC3B-II to autophagosomes and the formation of autophagic vacuoles (Figures 4B,C). These results are in agreement with the same results that were observed in shG9a cells (Figure 4E). The formation of LC3B-positive puncta was increased markedly after G9a was downregulated (Figures 4F,G). The activation of autophagy in shG9a cells was further supported by Western blot analyses (Figure 4H). Moreover, when the autophagic flux was inhibited by CQ, there were still a large number of vacuoles in cells, and cell counting results showed that the cell viability is worse repressed in the BIX-combined CQ group (Supplementary Figure 1D). This result indicated that G9a inhibition does not induce autophagic flux in GBM cells, but caused accumulation of autophagy (Klionsky et al., 2016). Together, our results provide evidence that G9a deficiency induced autophagy in glioblastoma cells.

### G9a Regulates C-Myc Expression to Control Glioblastoma Cell Proliferation

**Figure 5:**
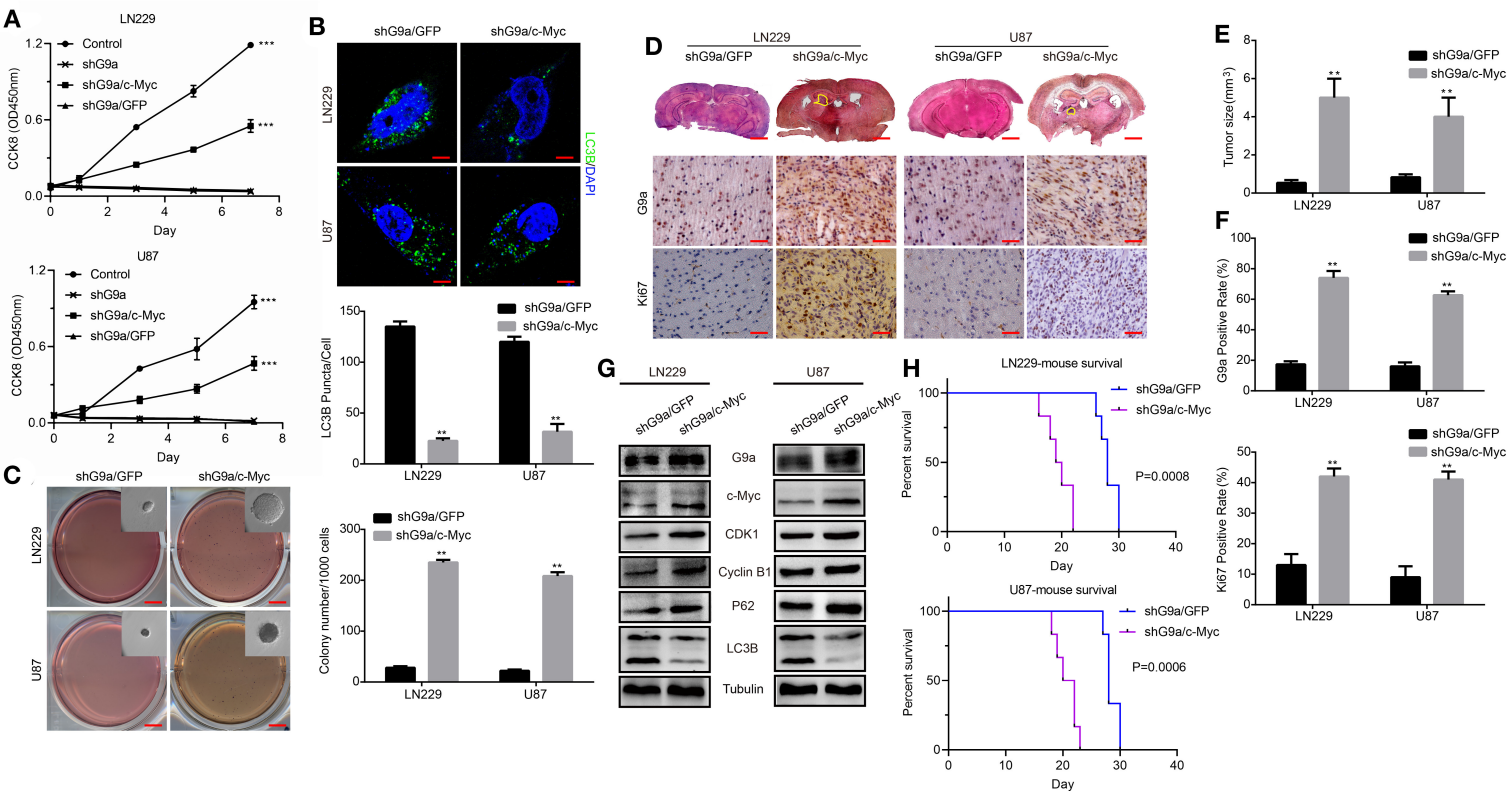
G9a regulates c-Myc expression to control glioblastoma cell proliferation. **(A)** CCK-8 assay was performed to examine the effect of c-Myc overexpression on cell proliferation of G9a-knockdown cells. c-Myc overexpression could rescue the proliferative ability. **(B)** (Upper) Fluorescence micrograph of immunofluorescence assay of LC3B expression. (Lower) Quantification of LC3B puncta in LN-229 and U-87 MG cells after c-Myc was upregulated in shG9a cells, Scale bar, 5 μm. **(C)** The colony formation assay was performed to examine the effect of c-Myc overexpression on colony formation ability of G9a-knockdown cells. **(D)** Orthotopic implantation was performed after c-Myc was restored in G9a-knockdown cells. Representative images of the hematoxylin and eosin (H&E) staining (upper) and immunohistochemistry analysis of G9a expression (middle) and Ki67 expression (lower) are presented. **(E)** Quantification of effects of c-Myc overexpression on tumor size. **(F)** G9a expression and Ki67-positive cells in G9a-knockdown tumor sample. **(G)** Western blot analysis of the correlation of expression of G9a and c-Myc, G2 cell cycle regulatory proteins, and autophagy-related genes in tumor sample after c-Myc was restored in G9a-knockdown cells. **(H)** Survival curve of mice in orthotopic transplantation. All experiments were carried out in triplicates independently, and all data are shown as the means ± s.d. **P < 0.01, ***P < 0.001. All *P*-values are based on control vs. treatment.

To gain more insight into the molecular mechanism of how G9a regulated cell proliferation and autophagy, we performed a microarray profiling and analyzed the data. Among the genes with significant differences in expression in U87 cells transduced with shG9a or shGFP, many oncogenesis-related genes were screened by the Gene Ontology (GO) functional term “oncogene” (Supplementary Figure 1E). We found that the expression of the oncogene c-Myc was one of these oncogenesis genes that were strongly associated with G9a. We carried out Western blot experiments to further confirm the expression relationship between G9a and c-Myc. The results showed that deficient G9a expression led to a remarkable decrease of c-Myc expression (Figures 4I,J). In addition, to further prove that G9a deficiency induced cell proliferation suppression and autophagy activation through the effect of c-Myc, we overexpressed c-Myc after G9a was silenced to examine whether this would reduce autophagy and rescue cell proliferation. CCK-8 assays demonstrated that overexpression of c-Myc rescued the cell proliferation of G9a-knockdown cells (Figure 5A). Soft agar colony formation experiments indicated that clonogenic abilities of shG9a cells successfully recovered after overexpressing c-Myc (Figure 5C). Immunofluorescence staining confirmed that the formation of LC3B-positive puncta was sharply dropped after c-Myc was upregulated in shG9a cells (Figure 5B). Furthermore, we validate our data *in vivo*, and the tumor growth was recovered in NOD/SCID mice injected with G9a-knockdown cells when c-Myc expression was upregulated (Figures 5D,E). Then, we found that Ki67 expression was also rescued in the tumor sample (Figure 5F). Consistently, Western blot assay (Figure 5G) showed that the expression of CDK1 and Cyclin B1 was restored in the c-Myc overexpression tumor sample accompanied by G9a and c-Myc protein expression level returning; meanwhile, autophagy was decreased after overexpression of c-Myc in the tumor sample. The expression of p62 was increased in the c-Myc overexpression tumor sample, and LC3B expression and the conversion of LC3B-I to LC3B-II were depressed. In addition, the survival time of mice was shortened after overexpression of c-Myc (Figure 5H). Indeed, as shown in Figure 5, c-Myc overexpression could restore cell proliferation and reduce autophagy in the G9a-knockdown cells, suggesting that the inhibition of cell proliferation and activation of autophagy mediated by knocking down G9a are due to the role of G9a in regulation of c-Myc expression in glioblastoma cells.

### G9a Binds to c-Myc Promoter and Activates Its Transcription

**Figure 6:**
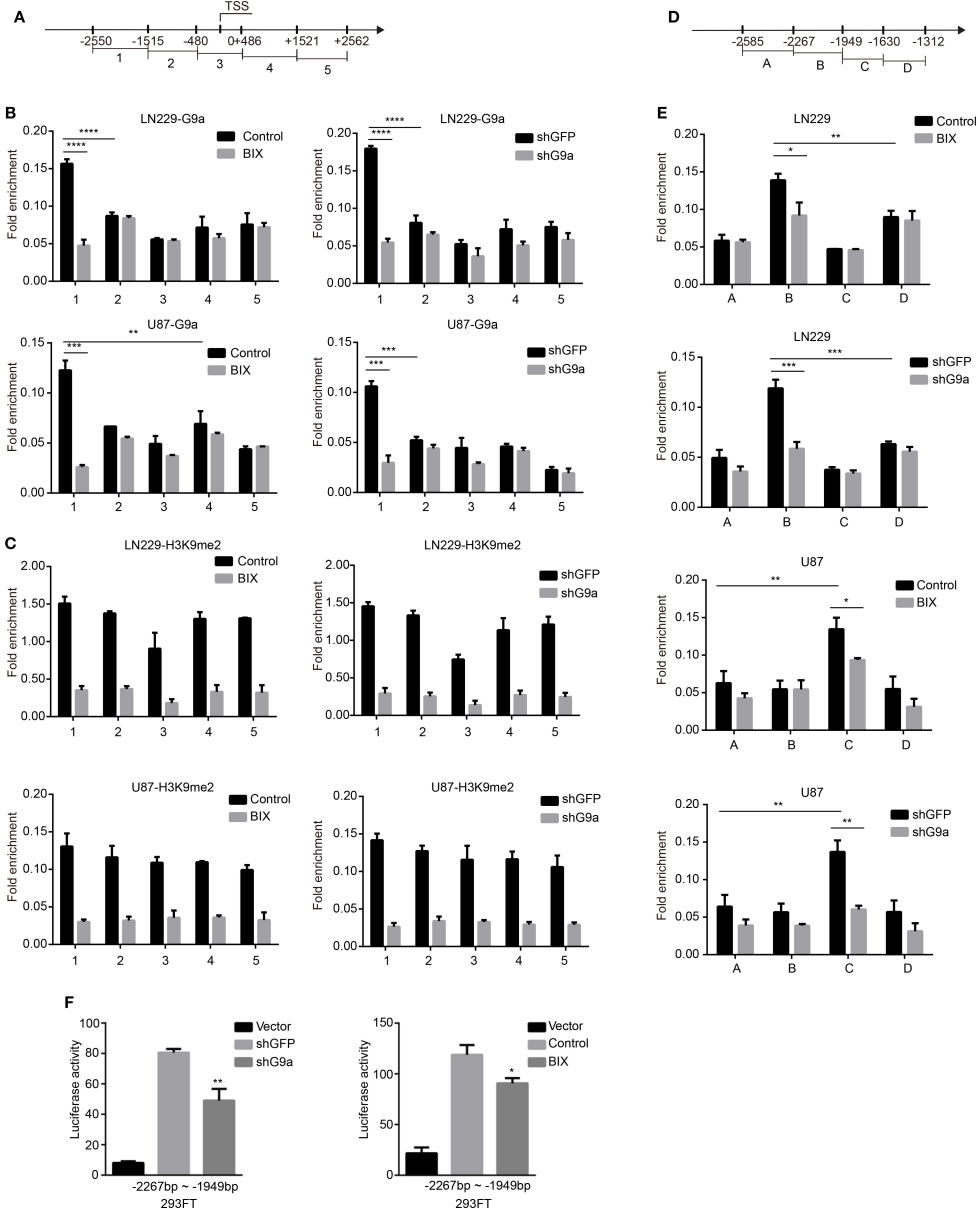
G9a binds to c-Myc promoter. **(A)** A total of five sets of primers were designed within the human c-Myc promoter. **(B)** A chromatin immunoprecipitation (ChIP) and quantitative real-time PCR (qPCR) (ChIP–qPCR) assay was performed using G9a antibodies to examine G9a enrichment at the promoter of c-Myc. **(C)** ChIP–qPCR assays were performed using H3K9me2 antibodies to examine H3K9me2 enrichment at the promoter of c-Myc. **(D)** A total of four sets of primers were designed within the −2550 to −1515 region of c-Myc promoter. **(E)** ChIP–qPCR assays were performed again using G9a antibodies to map the G9a-binding locus region. **(F)** Dual-luciferase reporter assays evaluating c-Myc promoter activity with G9a knockdown or inhibitor treatment. Luciferase activity was normalized with the pGL3-Basic vector. All the enrichment value was normalized by IgG. All experiments were carried out in triplicates independently, and all data are shown as the means ± s.d. *P < 0.05, **P < 0.01, ***P < 0.001, ****P < 0.0001. All *P*-values are based on control vs. treatment.

To further investigate how G9a regulated c-Myc expression in glioblastoma cells, we performed ChIP-qPCR assay to determine whether G9a could bind to the c-Myc promoter. A total of five sets of primers were designed (Figure 6A) related to five fragments within the c-Myc promoter region (−2250 to +2562 bp). As predicted, a significant enrichment of G9a was found at the −2550 to −1515 region of the c-Myc promoter. Then, the enrichment of G9a on this region was obviously decreased after G9a knockdown or inhibition (Figure 6B). Meanwhile, we also checked the enrichment of H3K9me2 on the c-Myc promoter region. While enrichment of H3K9me2 was also found at the promoter of c-Myc, there are no significant differences between the five fragments within the c-Myc promoter region. However, when G9a was downregulated or inhibited, the enrichment of H3K9me2 on the c-Myc promoter was sharply dropped, but there are still no significant differences between the five fragments (Figure 6C). These data provide evidence that G9a regulated c-Myc expression by directly binding to the c-Myc promoter. Furthermore, to map the G9a-binding locus region on the c-Myc promoter, we divided the −2550 to −1515 region of c-Myc promoter into four fragments and designed the corresponding primers (Figure 6D), and then performed ChIP-qPCR assay again. As shown in Figure 6E, G9a was enriched on the −2267 to −1949 region in LN-229 cells, and the −1949 to −1630 region in U-87 MG cells. Besides, after G9a knockdown or inhibition, the enrichment of G9a was visibly declined. To provide more relevant data, dual-luciferase reporter assays were applied. The −2267 to −1949 fragment was inserted into the pGL3 basic vector, and then the construct was co-transfected with the pRL-TK plasmid. The empty pGL3-basic vector was used as a negative control. The results shown in Figure 6F identified that the c-Myc promoter activity was significantly reduced after knockdown or inhibition of G9a.

Together, our results demonstrated that G9a modulates glioblastoma cell proliferation and autophagy by directly and transcriptionally activating c-Myc.

## Discussion

Malignant gliomas are reported as the third leading cause of cancer death for patients between the ages of 15 and 34, accounting for 2.5–4% of the global cancer death (Mallick et al., 2016; Hanif et al., 2017; Silantyev et al., 2019). In adults, glioblastoma represents more than half of all gliomas, and it is characterized by aggressive malignant phenotypes, poor prognosis, and low survival rates (Westermark, 2012; Omuro and DeAngelis, 2013). The 5-year survival rate of adult glioblastoma is of 2%, and pediatric glioblastoma is <20% (Hanif et al., 2017; Coleman et al., 2020). Currently due to the lack of effective target treatment available for glioblastoma, a good understanding of molecular regulation of this cancer is of key importance to develop new therapeutic strategies.

Recently, epigenetic dysregulations are reported as an important regulation mechanism in cancer initiation and progression (Costa-Pinheiro et al., 2015) and are also considered to play an emerging role in glioblastoma development (Gusyatiner and Hegi, 2018). Additionally, more and more evidences demonstrated that histone modifications in glioblastoma, mainly including histone acetylation and methylation, may determine the development of this cancer (Romani et al., 2018). H3K9 methylation and its methyltransferase G9a are also considered as a prominent mechanism in cancer cell biology and a potential therapeutic target in cancer treatment (Casciello et al., 2015; Janardhan et al., 2018; Cao et al., 2019; Monaghan et al., 2019). Although the inhibitor of G9a was reported to sensitize glioma cells to TMZ and to induce the differentiation of glioma stem-like cell, the detailed correlations between G9a and glioblastoma genesis remains to be further elucidated. In this study, we found that G9a knockdown or inhibition by its specific inhibitor BIX01294 induced significant suppression of cell proliferation *in vitro* and tumorigenicity *in vivo* and activated autophagy in glioblastoma cells. This controlling function of G9a was rescued by overexpression of c-Myc afterwards. These findings reveal a modulation role of G9a in glioblastoma tumorigenesis and support G9a as a potential therapeutic target of glioblastoma.

Glioblastoma exhibits highly aggressive features involving a high mitotic activity and great local invasiveness; this glial tumor infiltration at 1–2 cm from the original tumor mass prevents a total tumor resection after surgery and results in a high rate of tumor recurrence (Grobben et al., 2002). Aberrant alterations of cell cycle, which are closely related to the capability of proliferation (Sun et al., 2016), are a critical event in cancer cells (Chukkapalli et al., 2014), as well as in glioblastoma. Our data showed that G9a knockdown or inhibition significantly repressed glioblastoma cell proliferation and induced cell cycle arrest at G2 phase. We also found the decreased protein expression of regulators CDK1, CDK2, Cyclin A2, and Cyclin B1 in the G2 phase checkpoint. Besides, due to the close relationship between cell cycle and activity of cell proliferation, the same result was confirmed by colony formation assays. Moreover, a marked decline of migration and invasion was observed in both inhibitor-treatment cells and G9a-knockdown cells, while a visible suppression of tumorigenicity was found in intracranial injection mice of the G9a-knockdown group.

Accumulating evidence indicates that autophagy is a new therapeutic strategy in glioblastoma treatment (Ciechomska, 2018). Although it is reported as a double-edged sword in cancer therapy, autophagy is still a valuable target in glioblastoma (Taylor et al., 2018). According to recent research reports, there are two kinds of autophagy. One is called “protective” autophagy, which can desensitize cells to stressful situations such as nutrient starvation (Pratt et al., 2016). The other one is considered as prolonged autophagy, which results in an unwarranted form of cell death similar to necrosis, known as autophagic cell death (Shimizu et al., 2014). In glioblastoma, both inhibition of protective autophagy and activation of autophagic cell death are believed to possibly become a potential therapeutic treatment, such as the use of CQ or other novel autophagy inhibitors can increase the sensitivity of glioblastoma to Bevacizumab *in vitro* (Chakrabarti et al., 2016; Ren et al., 2016; Huang et al., 2018), and autophagic cell death has been seen throughout a combination of chemotherapy with TMZ and HDACi or other anti-cancer drugs (Chiao et al., 2013). We identified the initiation of autophagy after G9a knockdown or inhibition by observation of abundant collections of LC3B in the cytoplasm. Data of Western blot analyses demonstrated an increased conversion of LC3B-I to LC3B-II and a decline of p62 expression. Furthermore, we found that c-Myc expression was positively correlated with G9a expression, and knockdown or inhibition of G9a reduced the expression of c-Myc.

As a key oncogene deregulated in many cancer types, c-Myc was also identified to play a central role in regulating the formation and progression of glioblastoma (Wang et al., 2008; Ning et al., 2019). Importantly, our data showed that G9a was enriched on the −2550 to −1515 region of the c-Myc promoter in both U-87 MG and LN-229 cells and actually demonstrated G9a transcriptionally regulating c-Myc expression. It was reported that, in glioma cancer stem cells, c-Myc regulates cell cycle progression by controlling Cyclin D1 and p21^WAF1/CIP1^ (Wang et al., 2008). p21^WAF1/CIP1^, the inhibitor of CDKs including CDK1 and CDK2, was transcriptionally inhibited by c-Myc (Mitchell and El-Deiry, 1999). Additionally, Cyclin A2, whose expression was induced by c-Myc (Liao et al., 2000), was reported to interact with CDK1 and CDK2 to control G2/M entry (Woo et al., 2006). As we mentioned above, the expression of CDK1, CDK2, Cyclin A2, and Cyclin B1 was decreased after G9a knockdown or inhibition in both LN-229 and U-87 MG cells. The restoration of c-Myc rescued the expression of CDK1 and Cyclin B1 in tumor samples. These results suggested that G9a modulates glioblastoma cell cycle process probably by controlling the function of c-Myc in CDK and Cyclin expression. Importantly, we also found that c-Myc overexpression in G9a-knockdown cells could restore cell proliferation and reduce autophagy. All of our data indicated that G9a regulated glioblastoma cell proliferation and autophagy by transcriptionally activating c-Myc. However, the detailed mechanism of G9a that collaborates with c-Myc to regulate autophagy needs to be further studied, and would be part of our future research. In summary, our results provide evidence to support G9a as a potential therapeutic target in glioblastoma treatment strategy. However, we found that the relationship between G9a expression and overall survival of patient was not as significant as we expected based on data from existing online database (Supplementary Figures 1G,H). However, we thought that the complex correlation between G9a expression and patient's prognosis was caused by G9a playing an important role in many biological processes in mammals. The prognostic value of G9a was still needed to be evaluated by abundant clinic data. The key factor of future utilization of G9a in glioblastoma treatment was to find a way to specifically introduce drugs targeting G9a into glioblastoma tumors, avoiding normal cells.

## Conclusions

Here, we show that G9a is essential for glioblastoma carcinogenesis and reveal a probable mechanism of it in cell proliferation control. G9a knockdown or inhibition by its specific inhibitor BIX01294 led to the downregulation of c-Myc, which significantly repressed glioblastoma cell proliferation and tumorigenesis ability both *in vitro* and *in vivo*, and led to a cell cycle arrest in G2 phase, and activated glioblastoma cell autophagy. Mechanic studies showed that G9a directly binding to the promoter of c-Myc transcriptionally induced its expression, thereby promoting the expression of CDK1 and Cyclin B1, which contributes to cell cycle progression. In summary, our study shows that G9a modulates glioblastoma cell proliferation and autophagy by directly and transcriptionally activating c-Myc, indicating that G9a serves as a potential therapeutic target in glioblastoma.

## Data Availability Statement

The original contributions generated for the study are included in the article/Supplementary Materials, further inquiries can be directed to the corresponding author/s.

## Ethics Statement

The animal study was reviewed and approved by the Animal Care and Use Committee of Southwest University.

## Author Contributions

XK, RZ, and HC designed the study. XK and RZ performed the major experiments. XZ performed the animal experiments. LZ performed the statistical analysis. XK wrote the manuscript. HC revised the manuscript. All contributing authors read and approved the final manuscript.

## Conflict of Interest

The authors declare that the research was conducted in the absence of any commercial or financial relationships that could be construed as a potential conflict of interest.

## Supplementary Material

Supplemental Figure 1 **(A)** Flow cytometry assay for cell cycle in LN-229, U-87MG cells after G9a knockdown by shRNA. **(B)** Flow cytometry assay for cell apoptosis in LN-229, U-87MG cells after G9a knockdown by shRNA. **(C)** Real-time PCR analysis to characterize the expression of metastasis-related genes in G9a-knockdown cells or inhibitor-treated cells. **(D)** Micrograph of LN-229 and U-87 MG cells after G9a inhibitor treatment or inhibitor combined Chloroquine treatment, Scale bar, 10 μm. Cell counting analyses was performed to examine the effect of inhibitor treatment or inhibitor combined Chloroquine treatment on cell proliferation. **(E)** Heatmap of oncogenes significantly associated with G9a. **(F)** U-87MG cells were injected subcutaneously in NOD/SCID mice. Tumor growth was inhibited by G9a inhibitor treatment. The xenograft tumor volume was measured by caliper measurements, and the xenograft tumor weight was measured after it was removed. **(G)** Analysis of the relationship between G9a expression and overall survival of patients in UALCAN cancer database. **(H)** Kaplan–Meier analysis of progression-free survival in the R2: microarray analysis and visualization platform. All experiments were carried out in triplicates independently, and all data are shown as the means ± s.d. ^*^*P* < 0.05, ^**^*P* < 0.01, ^***^*P* < 0.001, ^****^*P* < 0.0001. All *P*-values are based on control vs. treatment.
