# Supplementary figures and images for: Deficiency of G9a Inhibits Cell Proliferation and Activates Autophagy via Transcriptionally Regulating c-Myc Expression in Glioblastoma
Source: Front Cell Dev Biol. 2020 Nov 27;8:593964. doi: 10.3389/fcell.2020.593964 (PMC7729084; doi:10.3389/fcell.2020.593964)

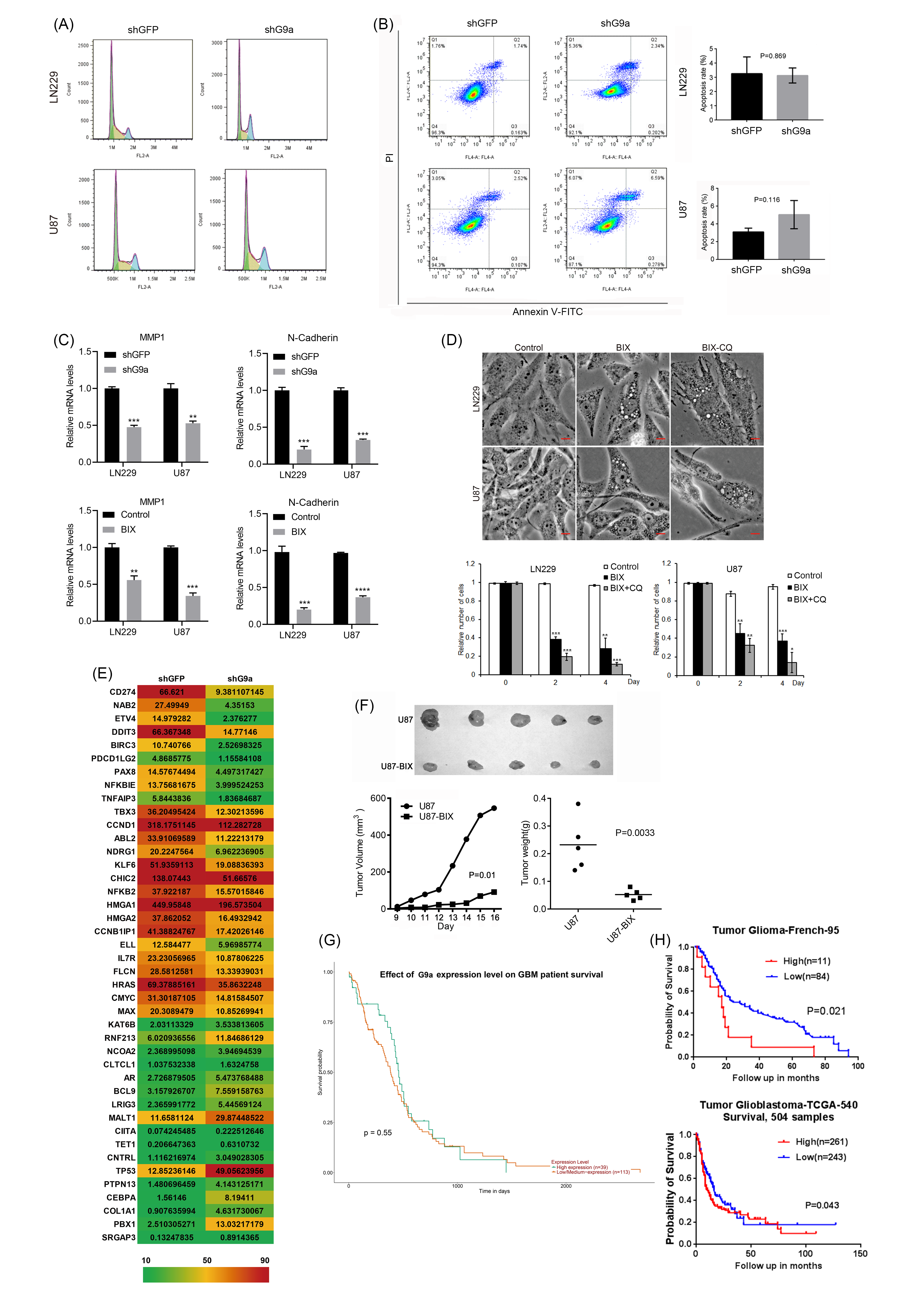

Supplement: Supplemental Figure 1 — (A) Flow cytometry assay for cell cycle in LN-229, U-87MG cells after G9a knockdown by shRNA. (B) Flow cytometry assay for cell apoptosis in LN-229, U-87MG cells after G9a knockdown by shRNA. (C) Real-time PCR analysis to characterize the expression of metastasis-related genes in G9a-knockdown cells or inhibitor-treated cells. (D) Micrograph of LN-229 and U-87 MG cells after G9a inhibitor treatment or inhibitor combined Chloroquine treatment, Scale bar, 10 μm. Cell counting analyses was performed to examine the effect of inhibitor treatment or inhibitor combined Chloroquine treatment on cell proliferation. (E) Heatmap of oncogenes significantly associated with G9a. (F) U-87MG cells were injected subcutaneously in NOD/SCID mice. Tumor growth was inhibited by G9a inhibitor treatment. The xenograft tumor volume was measured by caliper measurements, and the xenograft tumor weight was measured after it was removed. (G) Analysis of the relationship between G9a expression and overall survival of patients in UALCAN cancer database. (H) Kaplan–Meier analysis of progression-free survival in the R2: microarray analysis and visualization platform. All experiments were carried out in triplicates independently, and all data are shown as the means ± s.d. *P < 0.05, **P < 0.01, ***P < 0.001, ****P < 0.0001. All P-values are based on control vs. treatment. [file Image_1.TIF]
